# Stresses in the midpalatal suture in the maxillary protraction therapy: a 3D finite element analysis

**DOI:** 10.1186/s40510-016-0121-5

**Published:** 2016-03-16

**Authors:** Orlando M. Tanaka, Amando Yukio Saga, Matheus Melo Pithon, Marco Andre Argenta

**Affiliations:** School of Health and Biosciences, Pontifícia Universidade Católica do Paraná, Curitiba, Brazil; The Center for Advanced Dental Education, Saint Louis University, St. Louis, MO USA; School of Health and Biosciences, Pontifícia Universidade Católica do Paraná, Curitiba, Brazil; Southwest Bahia State University—UESB, Jequié, Bahia Brazil; Federal University of Paraná—UFPR, Paraná, Brazil

## Abstract

**Background:**

The aim of the present work was to evaluate the stress magnitudes and directions along the midpalatal suture in the maxillary protraction therapy.

**Methods:**

The geometry of the maxilla and teeth were digitally reconstructed based on computer tomography images obtained from the skull of a girl in a mixed dentition stage with skeletal and dental class III malocclusion. An appliance commonly used for rapid palatal expansion (RPE) was also digitally modeled for anchorage of the protraction force and meshed for finite element analysis. The maxillary protraction was simulated applying 600 cN (300 cN for each side) directed 30° forward and downward to the maxillary occlusal plane.

**Results:**

The principal stresses, through the force application, exhibited similar distribution patterns. A higher stress area was observed in the region of the midpalatal suture located in front of the incisive canal. All the sections showed vectors of compressive nature.

**Conclusions:**

Because of the compressive nature of the stresses distributed along the midpalatal suture in the maxillary protraction therapy simulation, which is opposite to the natural growth transversal tendency, maxillary expansion is advisable in clinical cases.

## Background

The skeletal class III malocclusion is caused by sagittal growth disharmony of the jaws that could occur due to underdevelopment of the maxilla, overdevelopment of the mandible, or a combination of both [1]. Basic treatment choices are growth modification, orthodontic camouflage, or orthognathic surgery depending on the patient’s age. Forward movement of the maxilla using an extraoral protraction force could correct the skeletal class III in children with retrognatic maxilla and apply tensile forces on the circum-maxillary sutures [2–7].

In animal models, it was histologically demonstrated that maxillary protraction therapy outcomes depend on the direction and magnitude of traction force [8, 9].

Class III patients often also exhibit maxillary transverse deficiency and, consequently, insufficient arch width [5, 6]. Based on the maxillary growth and ossification patterns, bone deposition in the midpalatal suture plays an important role in the progressive widening of the palate and alveolar arch [10]. However, a possible side effect of maxillary protraction is the possibility of mechanical constriction of the anterior region, since compressive strains were observed in the palate [11, 12].

Based on this, it is reasonable to hypothesize that maxillary protraction could generate a compressive stress environment in the midpalatal suture and that rapid palatal expansion would be necessary to compensate for this negative outcome and ensure normal development at midpalatal suture. Although several projects have examined the maxillary protraction by computation methods such as finite element analysis (FEA) [13–18], the specific mechanical benefit of maxillary expansion on the suture has not been demonstrated and questions remain on its necessity. Indeed, clinicians may sometimes opt to perform maxillary protraction with or without expansion. Also, there is no publication that has evaluated the mechanical environment considering the principal stresses.

Therefore, the purpose here was to mechanically compare both treatment modalities to provide basic scientific evidence on the stresses distribution along the midpalatal suture and provide a guide for the orthodontist to choose a more favorable protocol for skeletal class III maxillary protraction therapy.

## Methods

The geometry of the maxilla and teeth were digitally reconstructed basing on CT images acquired by cone beam computerized tomography (IS i-CAT®, Imaging Sciences, Hatfield, Pa) operated at 120 kVp, 0.5-mm nominal focal spot size, 14-bit dynamic range of gray scale, FOV of 126 mm, and 0.4-mm voxel size. A stack of 512 slices converted into exportable DICOM format files with thickness of 0.25 mm was produced. The CT images were obtained from the skull of a girl (age, 8.5 years) in a mixed dentition stage with skeletal and dental class III malocclusion.

**Fig. 1:**
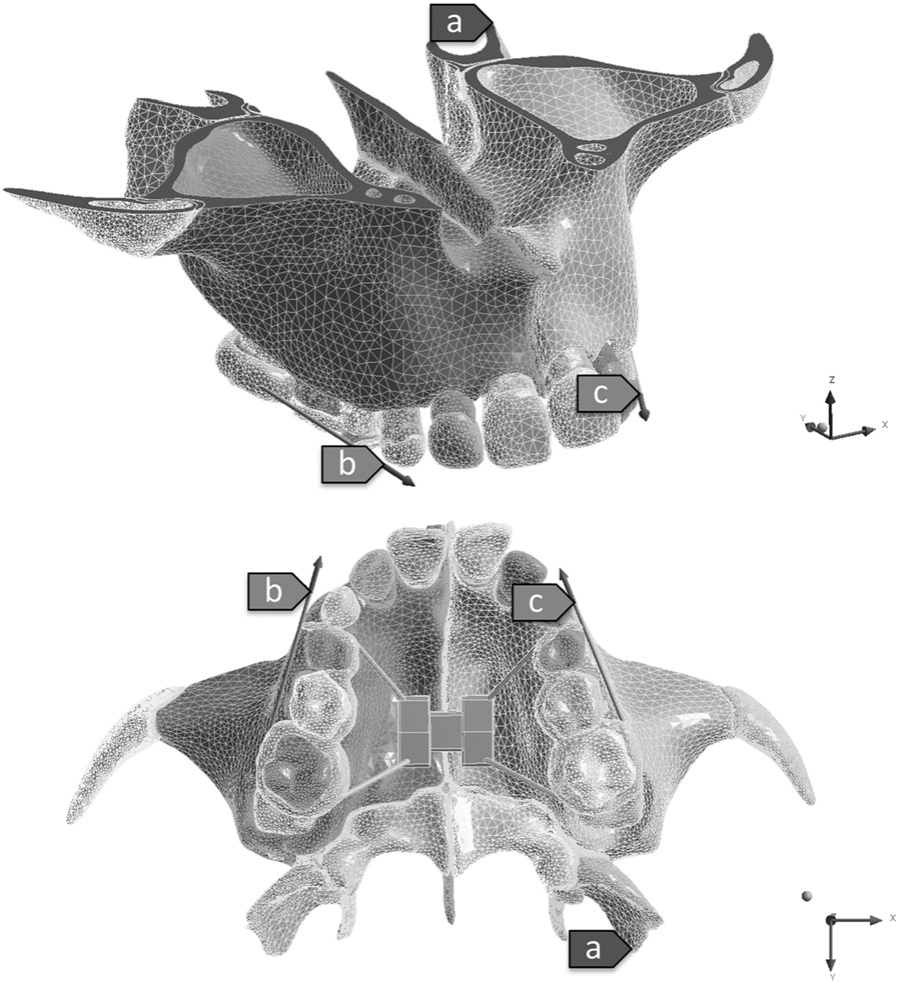
Model geometry. **a** Fixed support. **b**, **c** Protraction force

**Fig. 2:**
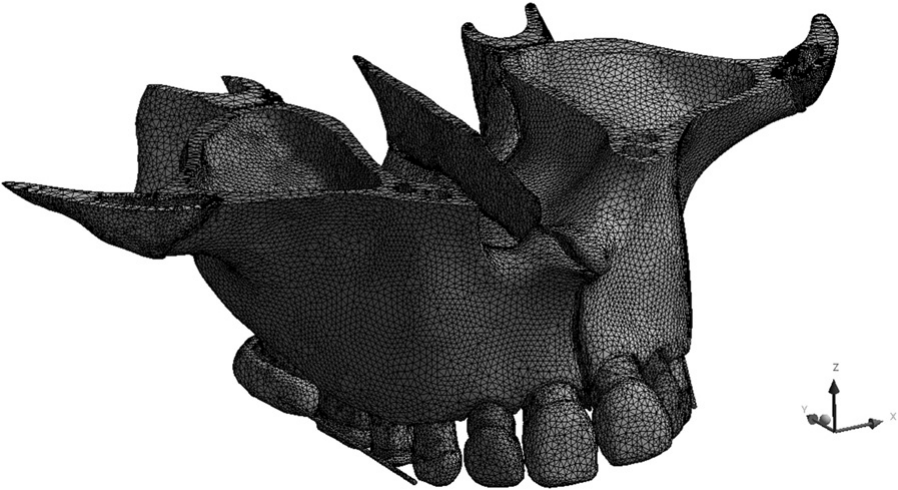
Finite element analysis mesh model

The tomographic slices were segmented to determine the limits of the cortical and trabecular bone layers as well as the enamel, dentin, and sutures. These limits were used to generate a solid 3D geometry and mesh using a computer-aided design software (Simpleware®, Innovation Centre, Exeter, UK). Hence, the anatomy of the generated model corresponded as closely as possible to the real anatomy of the skull. Since the study considered just the effects on the midpalatal suture, the maxilla was horizontally segmented in a plan above the nasal floor. A hyrax-type appliance commonly used for rapid palatal expansion (RPE) attached to the maxilla was digitally constructed with the sole objective of applying protractive force (Solidworks®, Dessault Systèmes Solidworks Corp., Concord, Ma) (Fig 1). The FEA simulated conditions of perfect union between considered structures. No activation of the appliance’s screw was simulated; since the purpose of the study was to analyze just the effects of the protraction loads in the midpalatal suture. The boundary condition was as follows: the pterygoid processes as fixed points, the upward and downward, forward and backward, and right and left displacement was constrained and it was imposed on zero-displacement and zero-rotation boundary conditions on the nodes along the horizontal segmented plan above the nasal floor (Fig 1). The experimental 3D model consisted of 573,726 tetrahedral elements sized ranging from 0.25 to 1.50 mm, 1,133,497 nodes, and 3,400,491 degrees of freedom (Fig 2).

The material properties of the elements representing tooth structures, alveolar bone, sutures, and stainless steel were assumed to be homogeneous, isotropic, and linearly elastic with specific Young’s moduli and Poisson’s ratios according to previous studies [19–23] (Table 1).

**Table 1:** Material’s mechanical properties used in this study

Material	Young’s modulus (MPa)	Poisson’s ratios
Cortical bone	13,800	0.26
Cancellous bone	345	0.31
Suture	0.68	0.47
Periodontal ligament	0.68	0.49
Enamel	84,100	0.20
Dentin	18,600	0.31
Stainless steel	200,000	0.30

The points of application of the protraction force in the palatal expansion appliance were positioned 3 mm buccally and anteriorly to the deciduous first molars crowns. The maxillary protraction was simulated applying 3 N for each side directed 30° forward and downward to the maxillary occlusal plane (Fig 1). Stress distribution in the midpalatal suture was analyzed using ANSYS (version 12.1, ANSYS Inc., Canonsburg, PA).

Numerical data produced color range maps of the principal stresses distribution, named maximum principal stress (MaxPS), middle principal stress (MidPS), and minimum principal stress (MinPS). Also, the results graphically demonstrate the directions of the principal stresses.

## Results

**Fig. 3:**
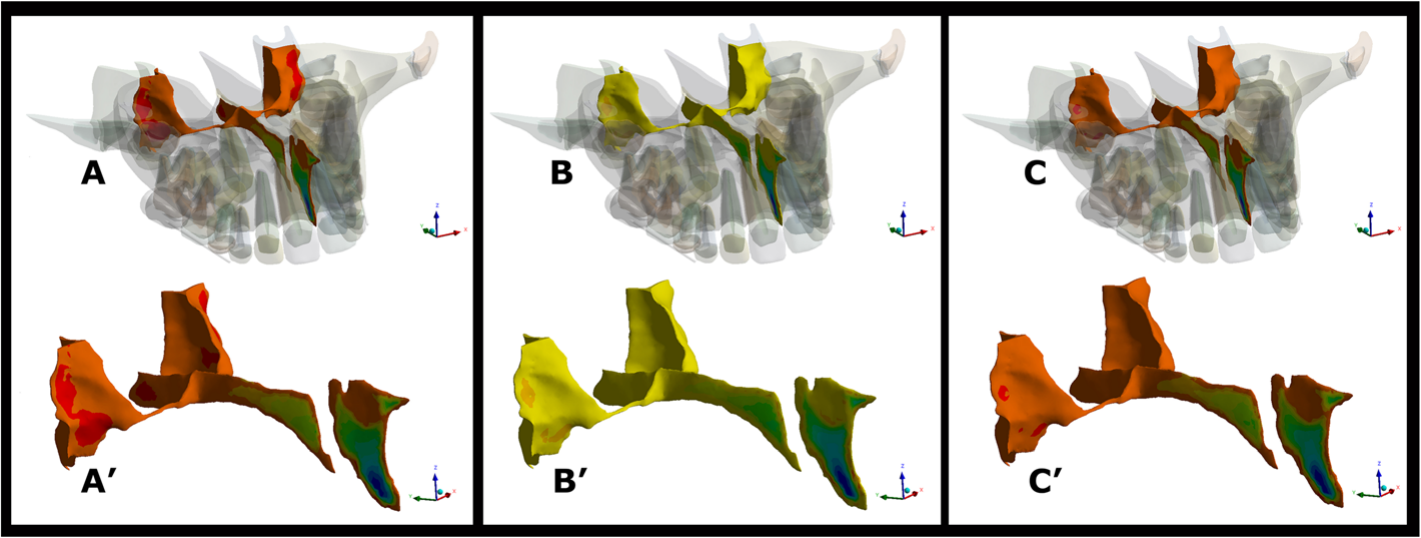
Principal stress distribution pattern. **a** MinPS—anatomic model view. **a**′ MinPS—isolated view of the midpalatal suture. **b** MidPS—anatomic model view. **b**′ MidPS—isolated view of the midpalatal suture. **c** MaxPS—anatomic model view. **c**′ MaxPS—isolated view of the midpalatal suture

The three principal stresses (MaxPS, MidPS, and MinPS) exhibited similar distribution patterns. A higher stress area was observed in the region of the midpalatal suture located in front of the incisive canal (Fig 3).

**Fig. 4:**
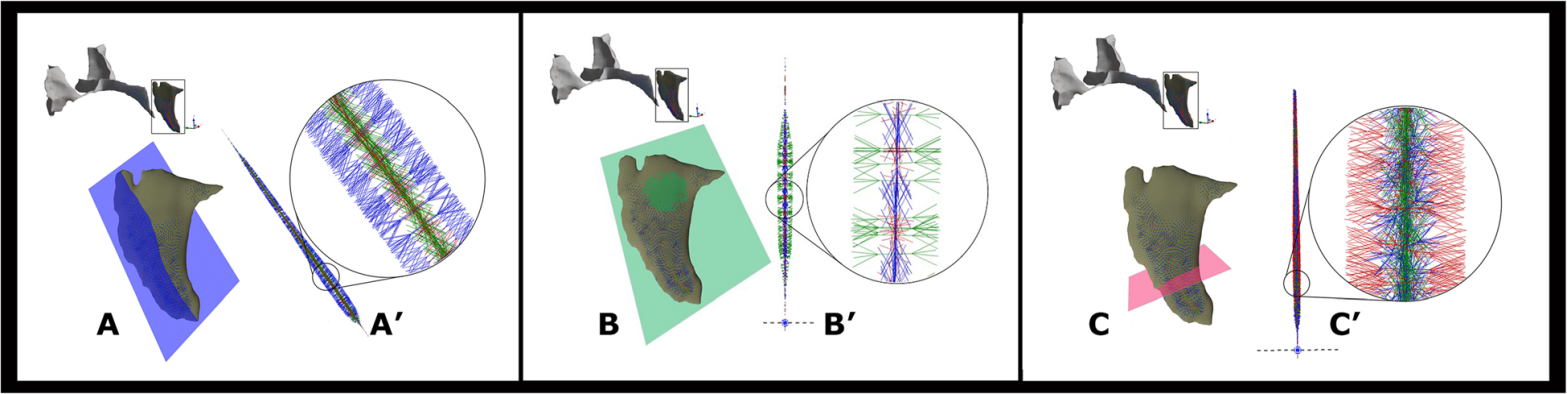
Principal Stress vector’s section. **a** MinPS—section position. **a**′ MinPS—enlarged view of the MinPS vectors’ directions. **b** MidPS—section position. **b**′ MidPS—enlarged view of the MidPS vectors’ directions. **c** MaxPS—section position. **c**′ MaxPS—enlarged view of the MaxPS vectors’ directions

Maximum and minimum stress values (kPa) of the MinPS, MidPS, and MaxPS for the loading model are given in the Table 2. Positive stress values express tension areas (warm colors), whereas negative values account for compression (cold colors) in the color range maps. To illustrate a detailed analysis of the directions of each principal stress, sections were obtained and the direction vectors were magnified (Fig 4). Because the area in front of the incisive canal presented the highest stress, it was emphasized in the figures. All the sections showed vectors, correspondent to the principal stresses, (MaxPS, MidPS, and MinPS) of compressive nature, and no tensile stress were observed.

**Table 2:** Maximum and minimum stress values observed (kPa)

Minimum principal stress		Middle principal stress		Maximum principal stress	
Max	Min	Max	Min	Max	Min
0.1054	−1.2262	0.1431	−1.1041	0.3347	−0.9740

## Discussion

Some benefits of the RPE in conjunction with maxillary protraction therapy in treating skeletal class III patients are correction of crossbites often associated with class III malocclusions, anchoring the maxillary dentition against forward movement and anterior constriction, and backward and downward rotation of the mandible [6, 24–27]. Turley [5] stated that palatal expansion “disarticulates” the maxilla and initiates cellular responses in these circummaxillary sutures, allowing for a more positive reaction to protraction forces potentiating orthopedic effects. Melsen [24] confirmed these increased cellular responses to RPE.

Kim et al. [28] in a meta-analysis of the effectiveness of protraction facemasks (based on 14 published articles) concluded that there were no distinct differences between treatments, with or without palatal expansion, except for maxillary incisor angulation, which increased in the nonexpansion treatment group. Similar results were also obtained by Vaughn et al. [17] that observed no statistically significant differences between groups with or without RPE in any measured variable. They also found no statistically significant differences in overall treatment time or in the time it took to achieve anterior crossbite correction and suggested that, in the absence of objective reasons for expansion such as maxillary width or space deficiencies, expansion will not aid the correction of class III malocclusions with facemask therapy.

Contrary opinion was supported by Yu et al. [18] using FEA to compare the amount of displacement and deformation of the maxilla, zygomatic arch, and circummaxillary sutures, dependent on whether the midpalatal suture was opened or not, showed there was a decrease in the upward–forward rotation of the maxilla and zygomatic arch with a greater amount of displacement in all-frontal, vertical, and lateral directions, when the midpalatal suture was opened, compared to when there was no opening of the midpalatal suture.

Gautam et al. [16] verified by FEA that the displacements of craniofacial structures were more favorable for the treatment of skeletal class III maxillary retrognathia when maxillary protraction was used with maxillary expansion. Hence, biomechanically, maxillary protraction combined with maxillary expansion appears to be a superior treatment modality for the treatment of maxillary retrognathia than maxillary protraction alone, in agreement with the present study. However, they did not present a comprehensive description of the mechanical environment within the suture (all principal stresses) and relied on von Mises stress values.

A tendency for constriction at the anterior region of maxillary arch had also been noted using different method approaches. Hata et al. [11], operating strain gauge transducer systems, analyzed the strain distribution and displacement of the human skull, and Tanne and Sakuda [12] observed the bones around the zygomaticomaxillary, frontozygomatic, and frontonasal sutures and more prominent compressive stresses with large compressive stresses perpendicular to the frontonasal suture plane.

The effects of micro-implant-assisted rapid palatal expansion showed tension and compression directed to the palate, while showing less rotation and tipping of the maxillary complex, and suggests that causes the maxilla to bend laterally, while preventing unwanted rotation of the complex [29], and by varying the location of N2 mini-implants and vector of class III mechanics, clinicians can differentially alter the magnitude of forward, downward, and rotational movement of the maxilla [30].

Our results present a comprehensive analysis of the principal stresses in the midpalatal suture. Since the maxillary expansion and protraction are generally performed in distinct phases and not simultaneously, this study focused only the protraction phase. Simultaneous simulation of the two phases could influence the distribution of stresses, confounding the results for protraction phase. We demonstrated that the potential for treatment benefits in performing RPE adjunctive to maxillary expansion definitely exists because the mechanical environment generated by protraction alone can disrupt or inhibit the normal growth of the midpalatal suture.

The constriction tendency was observed within all the sections of the suture, revealing that all principal stresses were of compressive nature during the maxillary protraction. Moreover, within the compressive stresses, negative on Table 2, the direction of the MinPS in the Fig. 4 a and a’ demonstrates that the highest compressions occur in the opposite direction to the transverse growth of the maxilla. This could aggravate the often already present transverse deficiencies in individuals with class III malocclusion. Hence, biomechanically, maxillary protraction combined with maxillary expansion appears to be a superior treatment modality for maxillary retrognathia than maxillary protraction alone. This forward and downward displacement of the nasomaxillary complex with maxillary protraction with expansion more closely approximates the natural growth direction of the maxilla [10]. Although previous clinical studies failed to provide a measurable benefit, our results show that lack of expansion could promote worsening of transverse relationships. Furthermore, there are potential problems in study design associated with clinical studies, such as power/sample size, patient age, variability of response, actual measurements performed, and other complicating factors that could have prevented significant findings.

Seemingly, the applied parameters did not represent perfectly the complex structure and behavior of the dental, bone and suture tissues. The developing of more detailed parameters is required, so that mathematical equations and computational models could mimic a real biological situation as closely as possible. Notwithstanding, it was assumed that this behavior idealization was suitable to describe theoretically the initial stress distribution of the midpalatal suture in the maxillary protraction therapy.

In the present study, the soft tissue surrounding the bones and teeth were not considered, since the limit definition still were not always possible in computer tomography images. While it is reasonable to assume that mechanical forces of the appliance will greatly overcome the soft tissue resistances, it would be beneficial to investigate the effects of the facial musculature and other soft tissues, and more progressive research with clinical identification of dynamic modeling is required to reinforce these conclusions.

## Conclusions

Because of the fully compressive nature of the stresses distributed along the midpalatal suture in the maxillary protraction therapy simulation, which is opposite to the natural growth transversal tendency, maxillary expansion is highly advisable in clinical cases. Adding expansion forces during maxillary protraction therapy can help to maintain a favorable growing environment in the midpalatal suture.
